# Evaluation of Fluence Correction Algorithms in Multispectral Photoacoustic Imaging

**DOI:** 10.1016/j.pacs.2020.100181

**Published:** 2020-04-18

**Authors:** Xuewen Zhou, Nima Akhlaghi, Keith A. Wear, Brian S. Garra, T. Joshua Pfefer, William C. Vogt

**Affiliations:** a Fischell Department of Bioengineering, University of Maryland, College Park, MD, 02742, United States; b Center for Devices and Radiological Health, U.S. Food and Drug Administration, Silver Spring, MD 20993, United States

**Keywords:** spectral coloring, oximetry, oxygen saturation, tissue phantoms, diffusion theory, Monte Carlo

## Abstract

Multispectral photoacoustic imaging (MPAI) is a promising emerging diagnostic technology, but fluence artifacts can degrade device performance. Our goal was to develop well-validated phantom-based test methods for evaluating and comparing MPAI fluence correction algorithms, including a heuristic diffusion approximation, Monte Carlo simulations, and an algorithm we developed based on novel application of the diffusion dipole model (DDM). Phantoms simulated a range of breast-mimicking optical properties and contained channels filled with chromophore solutions (ink, hemoglobin, or copper sulfate) or connected to a previously developed blood flow circuit providing tunable oxygen saturation (SO_2_). The DDM algorithm achieved similar spectral recovery and SO_2_ measurement accuracy to Monte Carlo-based corrections with lower computational cost, potentially providing an accurate, real-time correction approach. Algorithms were sensitive to optical property uncertainty, but error was minimized by matching phantom albedo. The developed test methods may provide a foundation for standardized assessment of MPAI fluence correction algorithm performance.

## 1 Introduction

Multispectral Photoacoustic Imaging (MPAI) is an emerging hybrid biomedical imaging modality that combines the high functional contrast of optics with the deep penetration of ultrasound [1]. By employing multiple optical wavelengths, absorption spectra can be measured, enabling mapping of chromophore concentrations including oxyhemoglobin (HbO_2_) and deoxyhemoglobin (Hb) as well as contrast agents (e.g. dyes, nanoparticles) [2]. Additionally, HbO_2_ and Hb can be used to compute blood oxygen saturation (SO_2_) maps. Many preclinical and clinical applications of MPAI have been reported, including tissue oximetry [[3], [4], [5]], cancer detection and diagnosis [[6], [7], [8], [9]], cerebrovascular imaging [[10], [11], [12]], surgical guidance [13], and tumor margining [14].

One critical technical challenge in MPAI is the corruption of measured absorption spectra due to “spectral coloring” artifacts caused by both spatial and spectral variations in local fluence within the tissue [[15], [16], [17]]. Photoacoustic signal is proportional to absorption coefficient as well as local fluence as evident from the well-known photoacoustic equation:

(1) $$P\left(\vec{x},\lambda \right)=\text{Γ}\mu_{a}\left(\vec{x},\lambda \right)\text{Φ}\left(\vec{x},\lambda \right)=\text{Γ}\mu_{a}\left(\vec{x},\lambda \right)\text{Φ}\left(\vec{x},\mu_{a}\left(\vec{x},\lambda \right),\mu_{s}^{'}\left(\vec{x},\lambda \right)\right)$$

where P is photoacoustic pressure amplitude, $\vec{x}$ is 3D spatial position, λ is wavelength, $\mu_{a}$ is absorption coefficient, $\mu_{s}^{'}$ is reduced scattering coefficient, and Φ is local fluence. Fluence generally decreases with depth in tissue and thus degrades image uniformity, but spectral variations in tissue optical properties also cause significant wavelength-dependent differences in fluence distribution that corrupt measured spectra. Absorption spectra are generally assumed to be linear combinations of signals from several chromophores, and spectral unmixing algorithms are used to solve for chromophore concentrations at each pixel, often using least-squares methods [18,19]. Clearly, if measured MPAI spectra differ from target absorption spectra (a performance characteristic we denote here as spectral recovery), MPAI measurement accuracy of spectrum-derived biomarkers such as SO_2_ or contrast agent concentration may be significantly degraded. Fluence correction can be performed on photoacoustic images by dividing P by Φ, thus yielding a more accurate estimate of $\mu_{a}$. As subsurface fluence Φ is difficult to measure, computational modeling represents an appealing technique for estimating fluence distributions.

Numerous methods of modeling light propagation in tissue have been proposed for performing fluence correction in MPAI systems. Because the Radiative Transfer Equation (RTE) is difficult to solve analytically, a common alternative is to use the diffusion approximation (equivalent to P_n_ approximation, where n = 1, 2 or 3 depending on truncation of spherical harmonic expansion) when the assumptions of isotropic photon scattering and time invariance are valid [20,21]. The P_1_ approximation is widely used in biophotonics, offering reasonable accuracy in the diffusive regime, and the delta-P_1_ approximation can further improve accuracy in the ballistic regime [[22], [23], [24]]. Both P_1_ and delta-P_1_ approximations can be solved numerically (e.g., using finite element methods) to obtain fluence, which is especially useful for studying complex heterogeneous media but carries high computational cost [[25], [26], [27]]. Analytical solutions of these models are possible for homogeneous media [24,28], and one-dimensional solutions of the P_1_ approximation have been used for MPAI fluence correction due to their simple implementation [29,30]. However, these models can potentially be inaccurate for MPAI if assumptions regarding source geometry are not satisfied.

An alternative approximation is the diffusion dipole model (DDM), which simulates the fluence distribution produced by a collimated pencil beam in a semi-infinite turbid medium as the response to a pair of point sources, with a source at a particular tissue depth and an imaginary source above the tissue surface [31]. This model is well studied and yields good accuracy far from boundaries and sources, but to our knowledge has not been previously applied as an MPAI fluence correction technique. Since a large, finite-area beam may be approximated as a sum of pencil beams, the DDM may be considered a Green’s function or impulse response that can be convolved with the incident beam geometry to compute the fluence produced by large beams used in MPAI systems. Another advantage is that DDM can easily produce fluence values at arbitrary points, most notably in planes offset from the beam center, which is relevant to clinical MPAI devices that use offset illumination-detection geometries [32]. Monte Carlo (MC) simulation has also been applied for photoacoustic fluence corrections [[33], [34], [35]], and may be considered the ‘gold standard’ method for simulating photon propagation and computing fluence in turbid media as it offers high accuracy in both ballistic and diffusive regimes and supports arbitrary tissue optical properties and geometries. However, MC-based fluence corrections can carry high computational cost. MC runtimes can be reduced through various methods such as GPU parallelization and scaling methods [36,37], although it is unclear whether such methods could be made fast enough to enable real-time or adaptive MPAI fluence corrections.

While these models can estimate fluence distributions in tissue, *a priori* knowledge of tissue optical properties and their spatial distribution is required, which is difficult to ascertain for real tissue. MPAI can be combined with independent tissue optical property measurements fed back into fluence models, such as diffuse optical tomography [38,39] or acousto-optics [40,41], but incorporating these methods increases system cost. Development of fluence correction methods that reduce or eliminate *a priori* specification of tissue optical properties remains an active research area and has included use of multiple illumination geometries [42,43], eigenspectral analysis [44], machine-learning based fluence quantification [45], monitoring dynamic changes in target absorption [46], differential contrast agent methods [47], and SNR-regularization [48]. Additionally, several models have been used in iterative minimization schemes to move towards adaptive fluence corrections that do not require *a priori* information, including finite element models [49], direct solutions of the RTE [50], Bayesian approaches [51,52], and adjoint radiance MC [53,54].

Fluence correction algorithms are a key design aspect of MPAI devices, and the wide variation in approaches implies potentially significant differences in performance. Fluence model accuracy, appropriateness for particular device illumination/detection geometries, and robustness to uncertainty in optical properties are not well understood. The availability of well-validated test methods for assessing algorithm performance would greatly facilitate MPAI device development and optimization, and as a result, improve diagnostic interpretation of preclinical and clinical MPAI data and support regulatory evaluation. Our group has previously developed test methods for image quality evaluation and SO_2_ measurement accuracy [[55], [56], [57]], but fluence correction performance was not considered. The purpose of this work was to develop best practices for evaluating and comparing performance of fluence correction algorithms. Towards this overall goal, our study objectives were to (1) develop and validate a novel, computationally-efficient DDM-based fluence correction, (2) evaluate spectral recovery and SO_2_ measurement accuracy of several fluence correction algorithms in tissue phantoms, and (3) evaluate and compare fluence correction robustness to uncertainty in tissue optical properties.

## 2 Methods

### 2.1 Fluence Correction Models

In this study, we developed and compared performance of three fluence correction algorithms: (1) a simple, 1D diffusion approximation (1D-DA), (2) a novel approach based on the diffusion-dipole model (DDM), and (3) Monte Carlo simulations including modeling of the ultrasound transducer in tissue contact (MC-UST). These three methods illustrate algorithms of varying complexity, ease of implementation, and computational cost. The 1D-DA algorithm may be considered a fast but very simple, even heuristic model, the DDM algorithm represents a more complex closed-form model tailored to our specific MPAI system configuration, and the MC-UST algorithm represents a highly detailed approach based on a gold-standard method.

#### 2.1.1 1D Homogeneous Diffusion Approximation (1D-DA)

For an infinite uniform planar beam in a semi-infinite homogeneous medium, assuming conservation of energy at the tissue surface and that fluence vanishes far from sources, the delta-P_1_ or delta-Eddington approximation can be used to solve for fluence as a function of depth, z:

(2) $$\text{Φ}\left(\text{z}\right)=H_{0}\left(1-R_{s}\right)\left[\alpha e^{-\left(\mu_{a}+\mu_{s}\left(1-g^{2}\right)\right)z}+\beta e^{-\mu_{eff}z}\right]$$

(3) $$\mu_{eff}=\sqrt{\;3\mu_{a}\left(\mu_{a}+\mu_{s}^{'}\right)\;}$$

where $H_{0}$ is incident radiant exposure, $R_{s}$ is surface specular reflectivity, $\mu_{s}$ is scattering coefficient, g is anisotropy factor, $\mu_{eff}$ is effective attenuation coefficient, and α and β are lumped parameters computed from tissue optical properties and surface reflectance conditions [24]. Fluence dependence on wavelength is left implicit here and in subsequent sections for clarity. The first term in brackets in equation 2 describes the collimated fluence while the second term describes diffuse fluence. The collimated term decays rapidly with depth such that its contribution to  Φ is less than 0.1% for depths greater than 4 mm, assuming typical tissue optical properties. Thus, a simple, heuristic one-dimensional diffusion approximation (1D-DA) can be derived as reported by others [15,29]:

(4) $$\text{Φ}\left(z\right)=\;H_{0}\left(1-R_{s}\right)\;e^{{(-\mu }_{eff}z)}$$

where $H_{0}\left(1-R_{s}\right)$ is the incident radiant exposure after specular reflection losses. While this heuristic model is quite simple and accurately models fluence directly beneath a large beam, its underlying assumptions are likely not well suited to predicting fluence distributions for our specific MPAI system configuration, which uses an offset beam-transducer geometry (see Section 2.2). However, a device designer could choose to use this approach due to its ease of implementation and very low computational cost. This algorithm is also fast enough to support real-time computations for adaptive or manually adjustable fluence compensation.

#### 2.1.2 Diffusion Dipole Model (DDM)

In this section, we briefly summarize our implementation of the DDM and refer the reader to Wang and Wu’s full description of this model [31]. Assuming a semi-infinite medium and using an extrapolated boundary condition [58], the fluence produced by a pencil beam, ${\text{Φ}}_{pencil}$, can be written in terms of fluence due to a point source within the tissue, ${\text{Φ}}_{point}$, and an imaginary point source above the tissue, ${\text{Φ}}_{point}^{*}$ [28,31]:

(6) $${\text{Φ}}_{pencil}(x,y,z)={\text{Φ}}_{point}(x,y,z)-{\text{Φ}}_{point}^{*}(x,y,z)=\frac{a^{'}}{4\pi D\rho_{1}}e^{-\mu_{eff}\left(\rho_{1}\right)}-\frac{a^{'}}{4\pi D\rho_{2}}e^{-\mu_{eff}\left(\rho_{2}\right)}$$

(7) $$\rho_{1}=\;\sqrt{{\left(x-x^{'}\right)}^{2}+{\left(y-y^{'}\right)}^{2}+{\left(z-z^{'}\right)}^{2}}$$

(8) $$\rho_{2}=\;\sqrt{{\left(x-x^{'}\right)}^{2}+{\left(y-y^{'}\right)}^{2}+{\left(z+z^{'}+2z_{b}\right)}^{2}}$$

where $\rho_{1}$ and $\rho_{2}$ are the distances from the real point source $\left(x',y^{'},z^{'}\right)$ and the imaginary point source $\left(x^{'},y^{'},-z^{'}-2z_{b}\right)$ to the observation point (x,y,z), $a^{'}=\mu_{s}^{'}/(\mu_{a}+\mu_{s}^{'})$ is the transport albedo, $D={\left(3\left(\mu_{a}+\mu_{s}^{'}\right)\right)}^{-1}$ is the diffusion coefficient, and $z_{b}$ is the location of extrapolated boundaries where fluence equals zero:

$$z_{b}=2D\frac{1+R_{eff}}{1-R_{eff}},\;{\;R}_{eff}=-1.440n_{r}^{-2}+0.710n_{r}^{-1}+0.668+0.0636n_{r}$$

where $n_{r}$ is the ratio of the tissue refractive index to the top medium (e.g., air). We set the real point source position at $x^{'}=y^{'}=0,\;z^{'}=1/(\mu_{a}+\mu_{s}^{'})$.

**Fig. 1:**
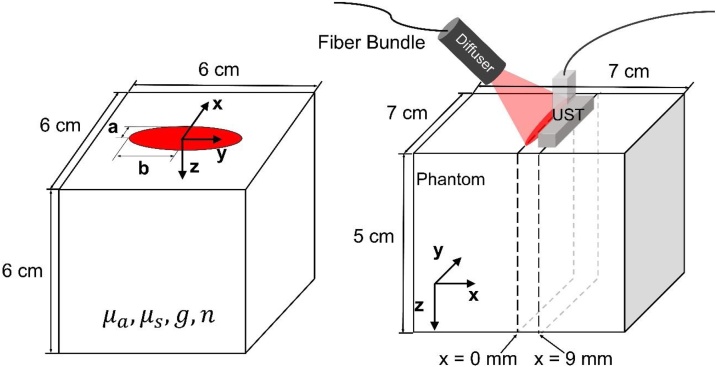
Left) Model geometry for DDM and MC verification simulations. Right) PAI system configuration used in phantom experiments and modeled by DDM and MC-UST fluence corrections.

Rather than pencil beams or point sources, however, most MPAI systems use large beams on the order of centimeters in length or diameter, including our custom system (see Section 2.2.1). In our approach, we convolve the above Green’s function with a uniform, normally-incident elliptical beam with minor radius a, major radius b, and radiant exposure $H_{0}$ [59] (Fig. 1):

(9) $${\text{Φ}}_{beam}\left(x,y,z\right)=\int_{-\infty }^{\infty }\int_{-\infty }^{\infty }{\text{Φ}}_{pencil}\left(x-x^{'},y-y^{'},z\right)S\left(x^{'},y^{'}\right)dx^{'}dy^{'}\;$$

(10) Sx',y'=H0, x'2a2+y'2b2≤10, otherwise 

where S describes the boundary of the elliptical beam. Because S is zero outside the ellipse, the integral bounds for equation (4) can be chosen as $x^{'}=\pm \;a$ and $y^{'}=\pm \;b$. The integral (equation 9) was computed using a 0.2 mm step size for $x^{'}$ and $y^{'}$, as convergence analysis yielded maximum residual error of < 1.3% near point sources between step sizes of 0.2 mm and 0.1 mm (data not shown). By fixing x equal to the elevational offset between the centers of the optical beam and ultrasound transducer and choosing y and z corresponding to MPAI pixel coordinates, the required 2D fluence correction map can be generated (Fig. 1). DDM fluence outputs were verified against MC simulations with $\mu_{a}$ = 0.1 cm^−1^, $\mu_{s}^{'}$ = 10 cm^−1^, g = 0.9, and n = 1.4, for four beam geometries with spatially uniform energy: 1) a 0.1 mm diameter point source, 2) a 4 cm diameter circular beam, 3) an elliptical beam with a = 0.5 cm, b = 2 cm, and 4) a 0.1 mm x 4 cm line source. Fluence error was spatially averaged over three regions in depth: shallow zone (0-0.5 cm), middle zone (0.5-3.0 cm), and deep zone (3.0-4.5 cm). Additional verification was performed for the case of a uniform elliptical beam (a = 1.75 cm, b = 0.25 cm) with various sets of tissue optical properties, specifically all combinations of $\mu_{a}=$ 0.02, 0.05, 0.1, or 0.2 cm^−1^, $\mu_{s}^{'}=$ 5, 10, 15, or 20 cm^−1^, and $n_{medium}=$ 1.4, 1.45, or 1.5. All MC simulations used for DDM verification assumed a normal incidence beam angle, matching the assumed condition of DDM and thus providing a true reference solution for comparison.

To investigate the potential to further reduce computational cost, we evaluated a one-dimensional DDM approximation (1D-DDM), where fluence vs. depth was computed at x = 9 mm, y = 0 mm (Fig. 1) and this 1D fluence distribution in depth was applied as a fluence correction to each column of the image data. 1D-DDM outputs were also compared against MC simulations.

#### 2.1.3 MC Simulations

A previously validated 3D voxel-based MC model was used to verify DDM algorithm accuracy and model the presence of the ultrasound transducer (denoted as MC-UST corrections) [60]. Based on prior convergence analysis, 8 × 10^7^ photons were launched into a 300 × 300 × 300 cubic grid with voxel dimensions of 0.02 × 0.02 x 0.02 cm. Unlike MC simulations used for DDM verification, MC-UST simulations assumed a 45° beam angle matching our MPAI system used in phantom experiments. MC-UST models used for fluence correction assumed an air boundary at the tissue surface ($n_{air}$ = 1.0), except for a rectangular area corresponding to the transducer face (12 mm x 40 mm) in contact with the tissue and positioned at a 9 mm offset from a 5 × 35 mm elliptical beam, mimicking our imaging configuration (Fig. 1). As our system’s transducer was covered with a gel-coupled aluminum foil layer to reduce surface clutter, we modeled photon interactions with the transducer using the Fresnel equations and a wavelength-dependent complex refractive index of aluminum [61]. Non-reflected photons are considered absorbed by the foil. Accurately modeling the transducer is a key consideration as transducer reflections have been shown to alter the fluence distribution in tissue [62] and transducer optical absorption can produce undesirable image clutter [32,56].

### 2.2 Phantom Experiments

#### 2.2.1 Custom PAI System

Phantom imaging experiments were performed using a custom MPAI system described previously [57]. In brief, the system uses a near-infrared optical parametric oscillator (OPO) delivering 5 ns pulses at a 10 Hz pulse repetition rate over a tunable wavelength range of 690-950 nm (Phocus Mobile, Opotek, Inc., Carlsbad, CA) and a 128-channel ultrasound acquisition system (Vantage 128, Verasonics, Inc., Kirkland, WA) with a 128-element, 7 MHz linear array transducer (L11-4v, Verasonics). Light is coupled into a fiber bundle and engineered diffuser to produce a 5 mm x 35 mm uniform elliptical beam positioned with a 9 mm offset relative to the transducer’s elevational center (i.e., 9 mm from the image plane). The transducer was gel-coupled with aluminum foil to reduce image clutter due to light absorption at the transducer surface. Multispectral imaging scans were performed from 700 to 898 nm in 2 nm steps, acquiring ten scans for averaging purposes, with wavelength-dependent radiant exposure of 8-10 mJ/cm^2^. Photoacoustic images were reconstructed in real-time using Verasonics’ proprietary pixel-based algorithm. MATLAB (Mathworks, Inc., Natick, MA) was used for all data acquisition and post-processing. MPAI data was fluence corrected using 1D-DA, 1D-DDM, or MC-UST algorithms, then least squares spectral unmixing using the pseudo-inverse approach [63] was applied to compute SO_2_ per pixel. Images acquired at eleven wavelengths were used for unmixing calculations (700, 720, 740, 760, 780, 800, 820, 840, 860, 880, 898 nm).

#### 2.2.2 Spectral Recovery

**Fig. 2:**
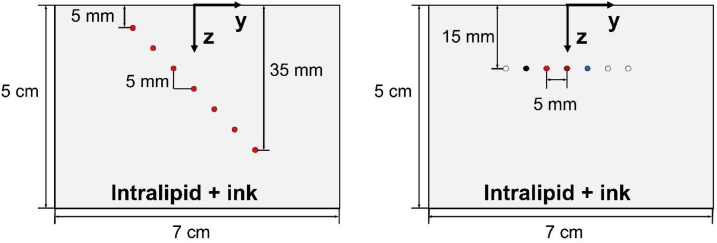
Cross-sectional view of phantom inclusion geometry for the penetration phantom (left) and chromophore phantom (right).

**Fig. 3:**
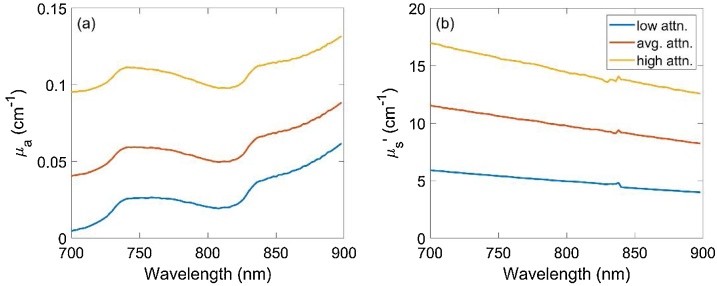
Measured absorption coefficient (a) and reduced scattering coefficient (b) spectra of Intralipid-ink phantoms with low, average, and high optical attenuation.

Phantom experiments were used to validate fluence models and evaluate spectral recovery using different fluence correction algorithms. Phantoms were constructed from acrylic molds with polytetrafluoroethylene (PTFE) tubes suspended at various positions and filled with various chromophore solutions. To characterize spectral recovery as a function of depth, a “penetration” phantom was constructed containing a diagonal array of PTFE tubes (0.56 mm inner diameter, STT-24, Component Supply Company, Fort Meade, FL) at depths of 5 to 35 mm in 5 mm steps with 5 mm horizontal spacing (Fig. 2). To improve validation as well as study robustness to uncertainty in tissue optical properties, three different Intralipid-ink solutions were used outside the tubes to represent low, average, and high optical attenuation of breast tissue at 800 nm: ($\mu_{a},\mu_{s}^{'}$) = (0.02 cm^−1^, 5 cm^−1^), (0.05 cm^−1^, 10 cm^−1^), and (0.1 cm^−1^, 15 cm^−1^), respectively. Phantom channels were filled with an India ink solution (Super Black India ink, Speedball Art Products Co., LLC, Statesville, NC) with $\mu_{a}=$ 4.5 cm^−1^ at 800 nm. Total transmittance and diffuse reflectance were measured over 400-1000 nm by integrating sphere spectrophotometry (Lambda 1050, PerkinElmer Inc., Waltham, MA) and the optical absorption coefficient and reduced scattering coefficient were computed using the inverse adding-doubling algorithm [64](Fig. 3).

To demonstrate adequate spectral recovery for a wide variety of target chromophore spectra, a second “chromophore” phantom was fabricated containing a row of seven tubes at a depth of 15 mm with 5 mm lateral spacing (Fig. 2). Four tubes were filled with either India ink, non-turbid hemoglobin solution (Multi-4 CO-Oximeter Control, Level 2, Instrumentation Laboratory, Inc., Bedford, MA), turbid bovine whole blood (Quad Five, Inc., Ryegate, MT), or copper sulfate (209198, Sigma-Aldrich Inc., St. Louis, MO). Solution concentrations were selected to yield absorption coefficients within 1.5 – 8 cm^−1^ over 700 to 900 nm, and blood was diluted to a total hemoglobin of 13.5 ± 0.1 g/dL. Reference extinction coefficient spectra of each solution were either measured by spectrophotometry or taken from the literature [65]. This phantom was filled with an Intralipid-ink solution representing the average attenuation case described above. Root mean square difference (RMSD) between the MPAI spectrum, $S_{corr}$, and reference spectrum, $S_{ref}$, over N= 11 wavelengths $\lambda_{i}$ was computed for each target tube as:

(12) $$RMSD=\sqrt{\frac{1}{N}\sum_{i=1}^{N}{\left(S_{corr}(\lambda_{i})-S_{ref}(\lambda_{i})\right)}^{2}}$$

Depth-averaged RMSD was also computed for penetration phantom data by averaging RMSD over all detectable target fluid channels. To quantify measurement variability, average spectral coefficient of variation (COV) was computed on uncorrected spectra for each target tube as:

(13) $$COV=\frac{1}{N}\sum_{i=1}^{N}\frac{\sigma_{i}}{\mu_{i}}$$

where $\sigma_{i}$ and $\mu_{i}$ are the standard deviation and mean of target intensity over ten scans, respectively, for the i -th scan wavelength.

#### 2.2.3 SO_2_ Measurement Accuracy and Robustness

To evaluate SO_2_ measurement accuracy of fluence correction models, we used our previously validated tunable blood oxygenation phantom approach [57]. Briefly, a penetration phantom was constructed with an array of tubes connected to a flow circuit that included a membrane oxygenator. By delivering adjustable concentrations of nitrogen and oxygen gas to the oxygenator, different blood SO_2_ levels can be achieved stably and reproducibly. Whole bovine blood was centrifuged to prepare red blood cell suspensions in phosphate buffered saline, and MPAI SO_2_ measurements were taken at setpoints of 99%, 80%, 60%, and 40% SO_2_. At each SO_2_ setpoint, a 0.2 mL blood sample was drawn from the circuit and measured using a clinical whole blood CO-oximeter (Avoximeter 4000, Accriva Diagnostics, Inc., San Diego, CA) to provide ground truth SO_2_ measurements both before and after MPAI measurements.

SO_2_ measurement accuracy was quantified using several performance metrics, including those described in an international performance standard for pulse oximeters [66] and in cerebral oximeter phantom test methods [67]. Sensitivity was defined as the slope of a linear fit to the SO_2_ data, and mean bias was computed as the mean difference between MPAI SO_2_ values, ${SO}_{2,\;PA,i}$, and CO-oximeter SO_2_ values, ${SO}_{2,\;CO,i}$, over M SO_2_ setpoints as:

(14) $$\bar{B}=\frac{1}{M}\sum_{i=1}^{M}\left(SO_{2,\;PA,i}-\;SO_{2,\;CO,i}\right)\;\;\;\;\;$$

RMSD is a more comprehensive metric that characterizes overall SO_2_ measurement performance and was computed for each target depth as:

(15) $${RMSD}_{SO2}=\sqrt{\frac{1}{M}{\sum_{i=1}^{M}\left({SO}_{2,\;PA,i}-\;{SO}_{2,\;CO,i}\right)}^{2}}$$

Again, depth-averaged ${RMSD}_{SO2}$ was computed to summarize performance over all target depths.

To evaluate the impact of uncertainty in tissue optical properties on SO_2_ measurement accuracy, MPAI data acquired in a penetration phantom with ($\mu_{a},\mu_{s}^{'}$) = (0.05 cm^−1^, 10 cm^−1^) and corrected using the 1D-DDM and 1D-DA algorithms were re-analyzed by varying input optical properties by $\text{Δ}\mu_{a}$ = −0.03 to +0.05 cm^−1^ in steps of 0.01 cm^−1^ and $\text{Δ}\mu_{s}^{'}$ = −3.0 to +5.0 cm^−1^ in steps of 1.0 cm^−1^. Depth-averaged ${RMSD}_{SO2}$ was computed for all combinations of $\mu_{a}+\text{Δ}\mu_{a}$ and $\mu_{s}^{'}+\text{Δ}\mu_{s}^{'}$.

## 3 Results and Discussion

### 3.1 DDM Verification

**Fig. 4:**
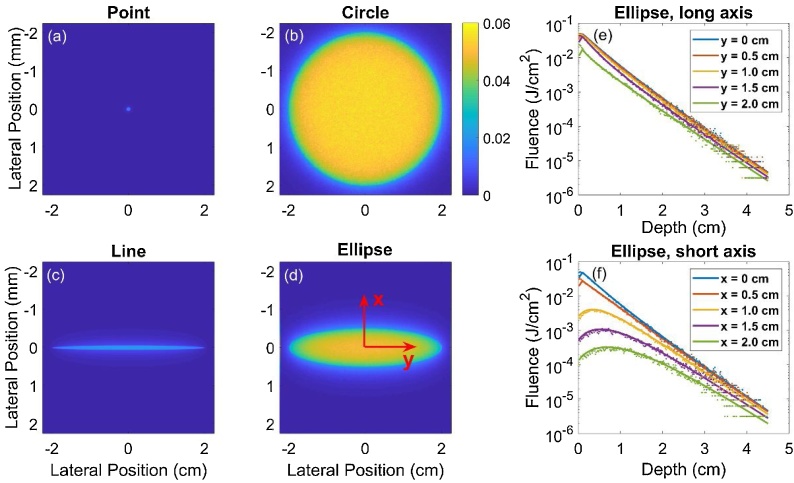
(a-d) En face images of the MC-computed fluence at the tissue surface for the four beam geometries. (e-f) Plots of DDM (solid line) and MC (points) fluence vs. depth for the elliptical beam geometry at different offset distances (0 - 2 cm) along the long axis (y) and short axis (x) of the beam.

DDM was verified against MC simulations with four commonly used beam geometries (Fig. 4). DDM and MC fluence values were generally in good agreement, and the lowest percent error was observed in the middle zone (Table 1). Greater error in the shallow zone is expected based on the limitations of diffusion theory near boundaries and sources, while errors in the deep zone are likely due to insufficient photon penetration and resultant noise in the MC outputs. Error in the shallow zone was generally higher for point and line beams, suggesting that the DDM spatial integration step value (optimized for the elliptical beam case, which is most relevant to our system design) should be decreased for these geometries.

**Table 1:** Average percent error in fluence between DDM vs. MC for the four beam geometries.

Source Geometry	Shallow Zone (0 – 0.5 cm)	Middle Zone (0.5 – 3 cm)	Deep Zone (3 – 4.5 cm)
Point (a = b = 0.05 cm)	47.1%	5.4%	34.5%
Circle (a = b = 2 cm)	6.0%	6.2%	21.1%
Ellipse (a = 0.5 cm, b = 2 cm)	4.5%	4.9%	24.5%
Line (a = 0.05 cm, b = 2 cm)	11.8%	5.2%	31.9%

**Fig. 5:**
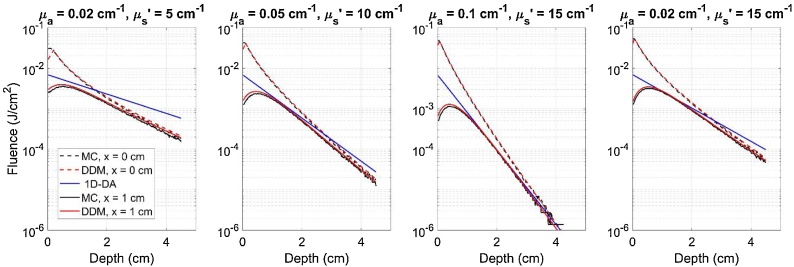
Fluence depth profiles beneath the beam (x = 0 cm) and in an elevational offset plane (x = 1 cm) computed with 1D-DA, DDM, and MC for the elliptical beam case and for four tissue optical property cases.

**Fig. 6:**
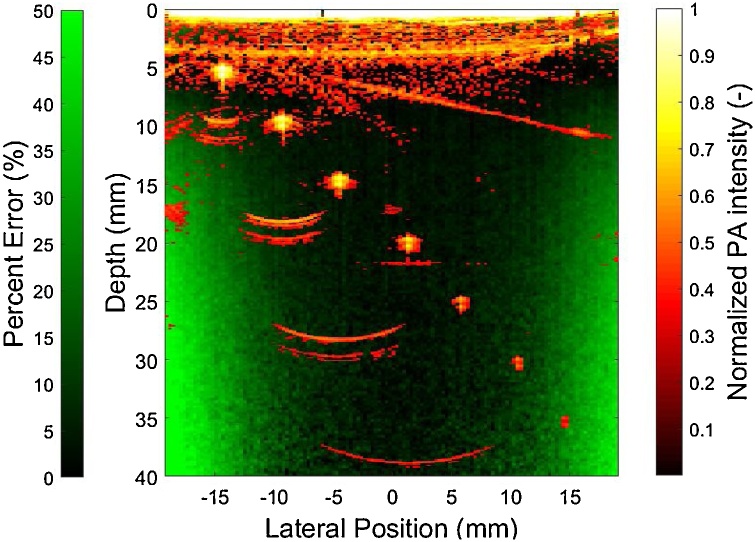
Overlay of a representative photoacoustic image acquired in the penetration phantom with low attenuation (red/yellow) and a map of percent error between 1D-DDM and MC fluence at an offset of x = 9 mm (green).

DDM was also in good agreement with MC simulations for the elliptical beam case, both at beam center and at offset planes useful for fluence correction in our MPAI system (Table 2). While the DDM and MC models can compute fluence in different offset planes, the 1D-DA model only produces a single fluence depth profile, indicating a lack of flexibility for modeling offset illumination-transducer geometries. As shown in Fig. 5, as tissue optical attenuation increases, the absolute error between 1D-DA and DDM/MC fluence increases significantly in the shallow zone. Similar results were found for the condition ($\mu_{a},\mu_{s}^{'}$) = (0.02 cm^−1^, 15 cm^−1^), which corresponds to the lowest absorption and highest reduced scattering coefficients used in verification studies. For a beam offset of 1 cm and for the high attenuation case, 1D-DA actually agreed better with DDM and MC results. However, results under other scenarios clearly indicate that this is not always the case. These results show that DDM is consistent with MC simulations and can predict fluence distributions in photoacoustic image planes offset from an elliptical laser beam. Comparisons of fluence between 1D-DDM and MC models showed that the regions of greatest error between 1D-DDM and MC fluence distributions are near the lateral edges of the field of view, with maximum errors exceeding 50% (Fig. 6). However, all but the deepest phantom targets were outside of these high-error zones as shown by the overlaid photoacoustic image in Fig. 6.

**Table 2:** Range of percent error between DDM- and MC-computed fluence in several depth ranges, averaged over multiple tissue optical property sets for the elliptical beam scenario.

	Y Axis			X Axis		
	Shallow Zone (0 – 0.5 cm)	Middle Zone (0.5 – 3 cm)	Deep Zone (3 – 4.5 cm)	Shallow Zone (0 – 0.5 cm)	Middle Zone (0.5 – 3 cm)	Deep Zone (3 – 4.5 cm)
Percent Error (%)	1.0 – 16.9	1.3 – 9.3	0 – 16.5	1.2 – 24.0	1.1 – 13.8	0 – 21.3

**Fig. 7:**
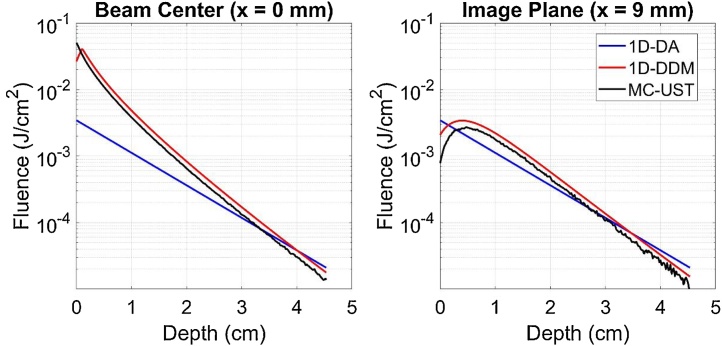
Fluence vs. depth predicted by each correction algorithm with $\mu_{a}=$ 0.05 cm^−1^, $\mu_{s}^{'}=$ 10 cm^−1^, both directly beneath the beam (left) and at a 9 mm elevational offset distance matching our MPAI system’s design (right).

As shown in Fig. 7, the 1D-DA algorithm predicts a significantly different log-linear slope of fluence versus depth than other models, with average percent error of 37% vs. both 1D-DDM and MC-UST. Agreement between 1D-DA and other algorithms improved somewhat at a 9 mm elevational offset corresponding to our system’s image plane, but average percent error remained large (31% and 22% vs. 1D-DDM and MC-UST, respectively). This improvement should be considered coincidental because the 1D-DA algorithm is only expected to be valid beneath the beam. At the beam center, the 1D-DDM and MC-UST fluence depth profiles in close agreement except from 0-2 mm (near the boundary and source). In the offset image plane, MC-UST predicts lower fluence than 1D-DDM for shallow depths (0-1 cm). This may be due to light absorption by the aluminum foil, which was included in the MC-UST model but not the 1D-DDM model.

### 3.2 Spectral Recovery

**Fig. 8:**
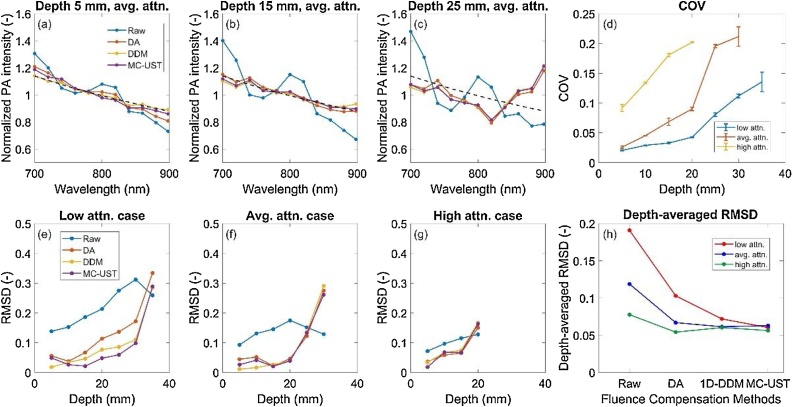
(a-c) Mean-normalized, fluence-corrected photoacoustic spectra vs. uncorrected spectra in targets at 5, 15, and 25 mm in the average attenuation phantom. Error bars omitted for clarity, with coefficient of variation (COV) plotted to convey signal variation over time. Black dashed lines represent the absorption spectrum of India ink from spectrophotometry. (d) COV of spectral measurements. (e-g) RMSD vs, target depth for all three phantom attenuation cases. (h) depth-averaged RMSD for the three attenuation cases.

**Fig. 9:**
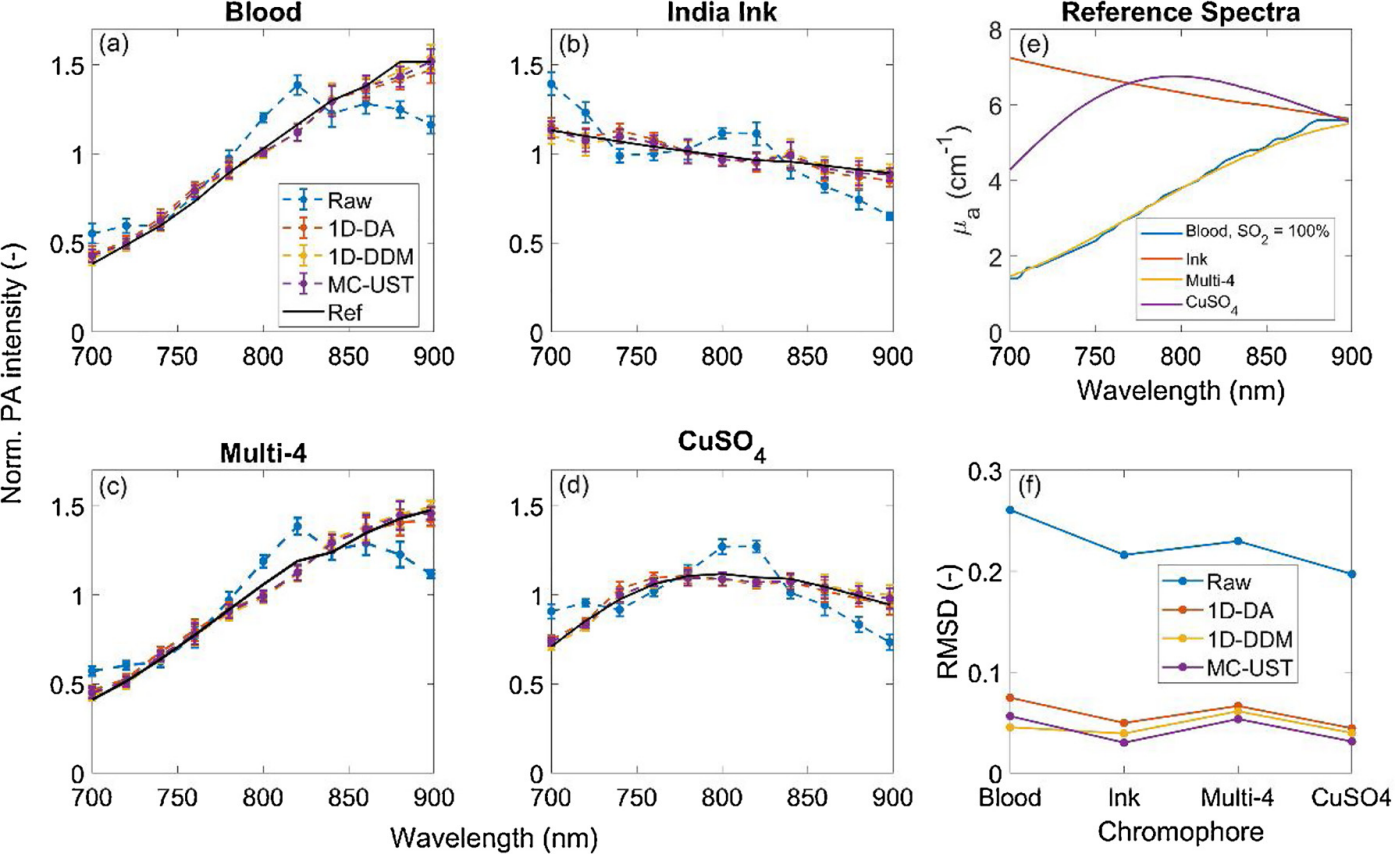
Measured photoacoustic (a-d) spectra and absorption (e) spectra of various chromophores, with comparative RMSD values (f).

Mean-normalized, fluence-corrected photoacoustic spectra acquired using each fluence correction algorithm were compared with the raw, uncorrected spectra (Fig. 8). Spectral RMSD and COV (equations 12 and 13) were analyzed and compared. All fluence correction methods improved recovery of the target spectrum, although spectral recovery generally decreased with depth. For different phantom optical property cases, the number of visible targets decreases with increasing attenuation as expected, but RMSD per target was comparable across algorithms provided measurements presented low enough COV. Qualitatively, the three fluence corrections provided comparable performance for all phantom optical property cases, but depth-averaged RMSD shows some slight difference between 1D-DA, 1D-DDM and MC-UST. The low attenuation case had the greatest depth-averaged RMSD since all seven targets were detectable and included in RMSD computations. Algorithm performance ranking in terms of RMSD was not consistent over all optical property cases, although the MC-UST algorithm’s RMSD was least variant with respect to phantom optical property cases (i.e., MC-UST was more robust). Small differences in RMSD between algorithms, especially for the high attenuation case, may be due to noise and may not indicate significantly different levels of performance. As shown in Fig. 9, fluence corrections accurately recovered spectra of various target chromophores. RMSD was low for all correction methods and similar across all chromophores imaged. This illustrates that fluence correction algorithm performance, in terms of spectral recovery, is generally independent of target absorption spectra presenting different magnitude and spectral features.

### 3.3 SO_2_ Measurement Accuracy and Robustness

**Fig. 10:**
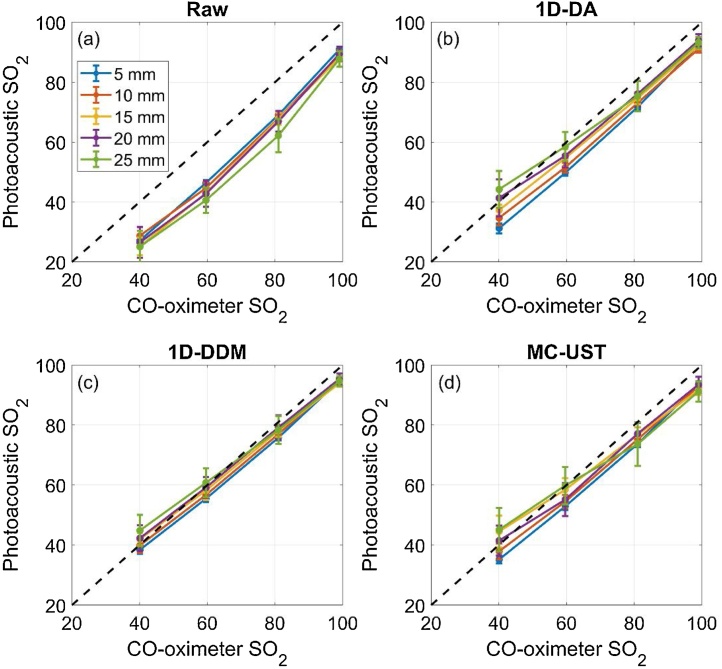
Photoacoustic vs. CO-oximeter SO_2_ measurements using a) no fluence correction, b) 1D-DA correction, c) 1D-DDM correction, and d) MC-UST correction.

**Fig. 11:**
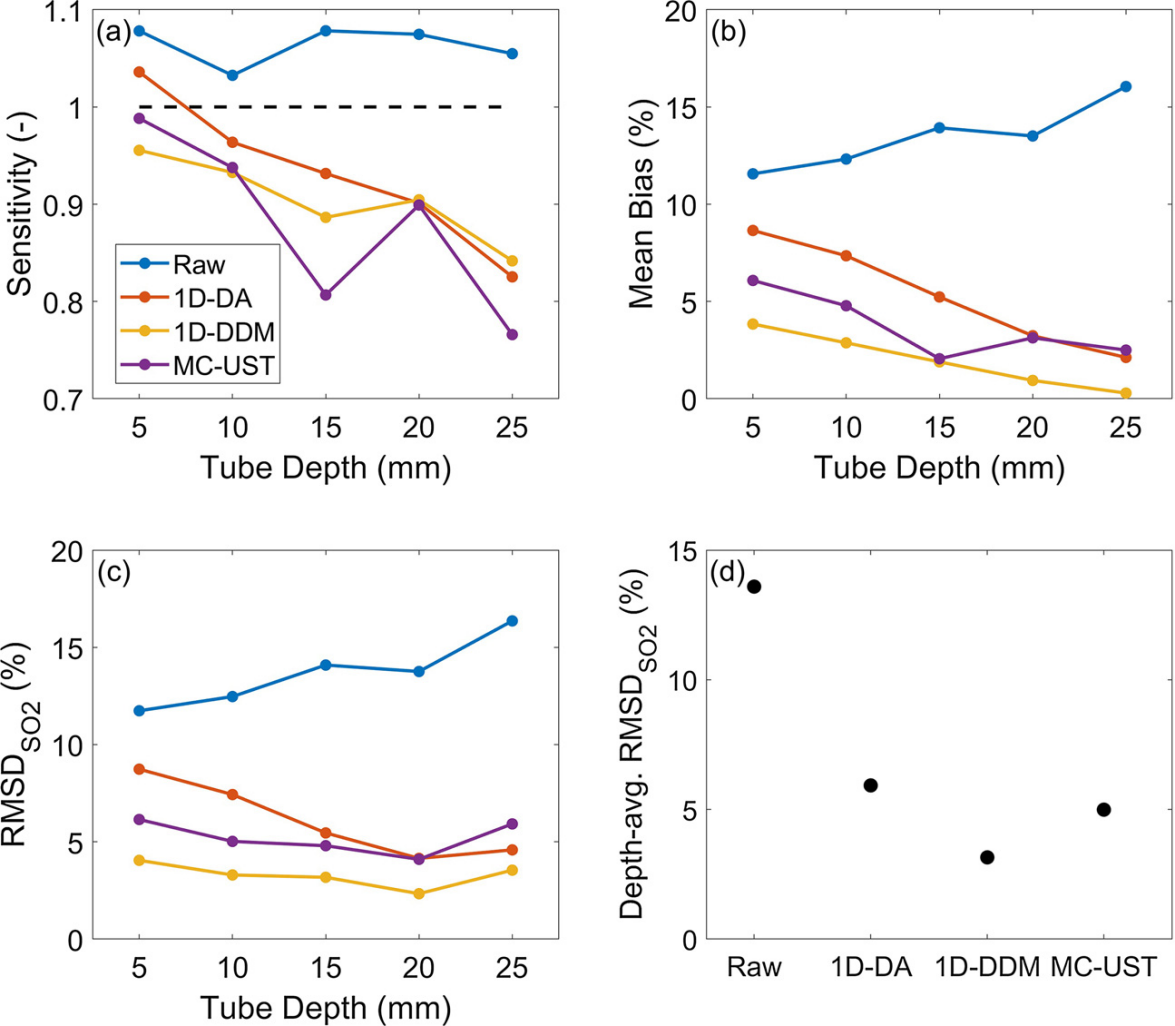
SO_2_ sensitivity (a), mean bias (b), and $RMSD_{SO2}$ (c) as functions of depth, as well as depth-averaged $RMSD_{SO2}$ (d) for each fluence correction algorithm. Black dashed line in (a) denotes the ideal sensitivity value of 1.

MPAI SO_2_ measurement accuracy varied significantly among fluence correction methods (Fig. 10), and MPAI measurements showed negative bias relative to CO-oximetry. SO_2_ sensitivity decreased with depth regardless of fluence correction algorithm, and the 1D-DA algorithm provided the most improvement in sensitivity (Fig. 11). However, the 1D-DA algorithm also produced the largest mean bias, particularly for shallow targets, which is consistent with fluence profiles shown in Fig. 5. If considering sensitivity and bias results alone, it is unclear how to rank algorithms by performance. We propose that $RMSD_{SO2}$ (equation 15) is a suitable metric for summarizing SO_2_ measurement accuracy, as applied in our previous work [57]. All algorithms improved $RMSD_{SO2}$ over performing no correction, but the 1D-DA algorithm had a strong depth-dependency due to inaccurate prediction of fluence attenuation rate (Fig. 11). The 1D-DDM algorithm achieved a depth-averaged $RMSD_{SO2}$ of < 4%, which is the acceptable performance threshold defined by ISO 80601-2-61:2017 for pulse oximeter measurement accuracy. 1D-DDM $RMSD_{SO2}$ was slightly lower than that achieved by the MC-UST algorithm, but this small difference may not be clinically significant. However, this result indicates that the simpler 1D-DDM model can achieve similar performance as the gold-standard MC approach with lower computational cost and ease of implementation.

**Fig. 12:**
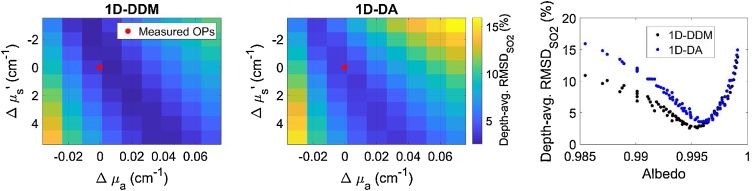
Depth-averaged $RMSD_{SO2}$ as a function of error in assumed tissue optical properties (OPs) relative to measured phantom properties for both 1D-DDM (left) and 1D-DA (center) algorithms. Right) Depth-averaged $RMSD_{SO2}$ as a function of varying assumed tissue albedo at 800 nm.

The requirement for *a priori* tissue property information is a significant limitation of many fluence correction algorithms for *in vivo* preclinical or clinical imaging, including those studied here. Results indicated that MPAI can achieve high SO_2_ measurement accuracy when the tissue optical properties are known *a priori*, but algorithm performance was highly sensitive to uncertainty in optical properties, with increases in $RMSD_{SO2}$ up to 15% (Fig. 12). Interestingly, we observed a region of the optical property tuning space that showed minimal changes in $RMSD_{SO2}$. This zone represents near-constant albedo, as confirmed by plotting depth-averaged $RMSD_{SO2}$ versus corresponding albedo for each pair of $\mu_{a}$ and $\mu_{s}^{'}$ values (Fig. 12). We also observed that our “true” optical property values measured by spectrophotometry were near this minimum, with small discrepancies attributable to measurement uncertainty. These results suggest that for models requiring *a priori* specification of optical properties, the design problem may be simplified to matching the tissue albedo, rather than matching both $\mu_{a}$ and $\mu_{s}^{'}$. This observation may inform design of future iterative or adaptive fluence correction strategies by reducing dimensionality of the fitting problem. These algorithms also assume a homogeneous medium, which may not be well-matched to *in vivo* conditions. Variations in tissue morphology and heterogeneity of optical properties are likely to affect accuracy and robustness to uncertainty of fluence correction algorithms, especially those investigated in this study. Additionally, variations in device design (beam position and angle, transducer face optical properties) may affect algorithm performance. These effects will be addressed in future work, for instance using multi-domain computational modeling [68].

## 4 Conclusions

We developed a novel, fast fluence correction based on the DDM and tailored for an offset illumination-detection geometry seen in clinical MPAI devices. This model was verified against Monte Carlo simulations and validated through phantom experiments. The 1D-DDM algorithm achieved similar spectral recovery and SO_2_ measurement accuracy to Monte Carlo-based corrections, but the 1D-DDM method offers lower computational cost and may enable real-time or adaptive fluence corrections. Assumption of a homogeneous medium and requirements for *a priori* assumption or measurement of tissue optical properties remain significant limitations of fluence correction algorithms evaluated in this study. While algorithm performance was sensitive to uncertainty in tissue optical properties, error was small when the simulated albedo was close to the true phantom albedo. This observation may aid design of future real-time, adaptive fluence correction strategies. Future work will include parametric study of algorithm robustness to uncertainty in device design parameters as well as tissue properties and morphology. The performance test methods and metrics proposed in this work may facilitate standardization of best practices for evaluating fluence correction algorithm performance in MPAI devices.

## Transparency document

Transparency document

## Declaration of Competing Interest

The authors declare that there are no conflicts of interest.
