# Supplementary material for: Evaluation of Fluence Correction Algorithms in Multispectral Photoacoustic Imaging
Source: Photoacoustics. 2020 Apr 18;19:100181. doi: 10.1016/j.pacs.2020.100181 (PMC7210453; doi:10.1016/j.pacs.2020.100181)
Supplement: Transparency document [file mmc1.zip › PACS_COI_Wear.pdf]

ELSEVIER

Photoacoustics

Conflict of Interest Policy

Manuscript number (if applicable):
Article Title: Evaluation of Fluence Correction Algorithms in Multispectral Photoacoustic Imaging

Author name: Keith A. Wear

Declarations

Photoacoustics requires that all authors sign a declaration of conflicting interests. If you have nothing to declare in any of these categories then this should be stated.

Conflict of Interest

A conflicting interest exists when professional judgement concerning a primary interest (such as patient's welfare or the validity of research) may be influenced by a secondary interest (such as financial gain or personal rivalry). It may arise for the authors when they have financial interest that may influence their interpretation of their results or those of others. Examples of potential conflicts of interest include employment, consultancies, stock ownership, honoraria, paid expert testimony, patent applications/registrations, and grants or other funding.

Please state any competing interests

I have no conflicts of interest to disclose.

Funding Source

All sources of funding should also be acknowledged and you should declare any involvement of study sponsors in the study design; collection, analysis and interpretation of data; the writing of the manuscript; the decision to submit the manuscript for publication. If the study sponsors had no such involvement, this should be stated.

Please state any sources of funding for your research

FDA Office of Women's Health
FDA Medical Countermeasures Initiative

Signature (a scanned signature is acceptable, but each author must sign)

Keith A. Wear -S Digitally signed by Keith A. Wear -S
Date: 2019.12.20 09:19:28 -05'00'
____

Print name

Keith A. Wear____ ____
